# Optimizing Palliative Cancer Surgery Trial Completion: Lessons Learned From Qualitative Content Analysis of S1316 – Comparative Effectiveness Trial for Malignant Bowel Obstruction

**DOI:** 10.1177/10499091251391420

**Published:** 2025-10-29

**Authors:** Mohammad Saad Farooq, Neda Amini, Virginia Sun, Gary B. Deutsch, Jeremiah L. Deneve, Marcia Grant, Kathryn B. Arnold, Angeles Alvarez Secord, Garnet Anderson, Robert S. Krouse

**Affiliations:** 1 Division of Endocrine and Oncologic Surgery, 21798 Hospital of the University of Pennsylvania, Philadelphia, PA, USA; 2 Department of Surgery, 21599 Geisinger Medical Center, Danville, PA, USA; 3 Division of Nursing Research and Education, Department of Population Sciences, 20220 City of Hope Comprehensive Cancer Center, Duarte, CA, USA; 4 Division of Surgical Oncology, 24998 NYU Langone Hospital - Long Island, Mineola, NY, USA; 5 Division of Surgical Oncology, University of North Carolina, Chapel Hill, NC, USA; 6 SWOG Statistics and Data Management Center, 7286 Fred Hutchinson Cancer Center, Seattle, WA, USA; 7 Division of Gynecologic Oncology, Duke Cancer Institute, Duke University Health System, Durham, NC, USA; 8 Division of Public Health Science, 7286 Fred Hutchinson Cancer Center, Seattle, WA, USA; 9 Department of Surgery, University of Pennsylvania, Leonard Davis Institute of Health Policy, Cpl. Michael J. Crescenz Veterans Affairs Medical Center, Philadelphia, PA, USA

**Keywords:** malignant bowel obstruction, palliative surgery, clinical trial, SWOG S1316, qualitative analysis, palliative care, MBO

## Abstract

**Background:**

Malignant bowel obstruction (MBO) is a complex clinical entity and there remains a relative lack of high-quality comparative trials on surgical management, in part due to a heterogeneous patient population and different treatment modalities which contribute to challenges in trial design and completion. SWOG S1316 is the only prospective randomized trial evaluating surgical vs non-surgical management of MBO and involved a trial framework in which patients were recruited for a randomization pathway as well as a patient choice pathway. Importantly, successful completion of S1316 required numerous amendment modifications to the trial during its course. We aimed to highlight aspects of S1316 trial design, execution, and modification that potentially contributed to trial completion.

**Methods:**

Iterative qualitative content analysis of trial modification amendments through the course of the trial from 2015 to 2020.

**Results:**

133 unique amendments were made to S1316 from 2015 to 2020. We found four dominant domains for the amendments: Accrual Barriers, Study Design Changes, Data Collection Issues, and Clarifications. Accrual amendments were essential to completing the trial and included increasing participating sites from six to 30 (including international sites) and the inclusion of Spanish-speaking participants (11% of final study population).

**Conclusions:**

Content analysis of S1316 trial amendments highlighted that Accrual amendments were important in trial completion. Future investigators may benefit from better anticipating trial modifications as they design their studies. It is likely that rapid initiation of trial amendments can lead to improved accrual and study completion.

## Introduction

Malignant bowel obstruction (MBO) is a complex, heterogeneous clinical entity with a significant global prevalence that imparts a challenging burden on patients and healthcare systems alike.^1,2^ The main treatment approaches are surgical or non-surgical, which may include endoscopic procedures.^1-3^ Despite a growing need for standardized practice guidelines on the appropriate management of MBO, there has remained a lack of prospective trials that can help guide clinicians to optimize patient-centered outcomes, in part due to complexity of trial design and completion in this patient population.^4,5^

The aforementioned gap in the palliative care literature was highlighted over 20 years ago, with clinicians/researchers stressing the importance of prospective investigation.^6^ Since then, SWOG S1316 is the only prospective randomized trial evaluating surgical vs non-surgical management of MBO, doing so by utilizing a novel pragmatic comparative effectiveness framework.^7^ S1316’s study design is particularly unique as it involved a “randomization” pathway, in which 56 patients were assigned surgical treatment (specific operation was at the discretion of the primary surgical team) vs medical treatment (bowel rest, supportive care, and somatostatin analogue therapy recommended), as well as a “patient choice” pathway, which prospectively analyzed 165 patients based on the treatment of their choosing (65 surgical, 100 medical). Key findings from S1316 included no difference in the primary outcome of “good days” (defined as days out of the hospital and alive at 91 days), but the trial did report improvements in patient MBO-specific quality of life metrics for patients managed with surgical treatment.^7^

To ensure completion, S1316 required numerous modifications throughout the course of the trial, prompting multiple amendments to the study. Amendments were prompted for multiple reasons, including but not limited to accrual delays, recurrent Spanish-only speakers that would have been eligible for participation, questions on clarifications or study changes from site coordinators/clinical investigators, and study team recognition of necessary clarifications. This report presents an innovative qualitative content analysis of multiple amendments needed to ensure study completion of this Agency for Health Research and Quality (AHRQ) supported and National Cancer Institute (NCI) funded NCI Community Oncology Research Program (NCORP) study. To date, this type of analysis has not been conducted for surgical clinical trials and, moreover, general qualitative content analysis in the surgical field is lacking.^8^ The primary purpose of this report is to provide practical information to guide clinicians and scientists as they develop future multi-site clinical trials for patients with advanced cancer, including those with disparate modalities of treatment.

## Methods

For this report, we analyzed multiple protocol amendments to S1316 implemented prior to the end of study accrual. Each individual amendment was collated in a Microsoft Excel file. A structured content analysis was then performed, and individual domains and themes emerged. Four members of the research team (MSF, MG, RK, NA) coded each amendment using a qualitative content analysis approach^9,10^. Our team included highly experienced qualitative researchers (MG, VS, and RK); others that coded were experienced clinical researchers (MSF and NA). Creation of the four main domains was an iterative process based on review of the amendments, with input from all team members to arrive at the final categorizations. Coding was initially performed independently, and then as a team, to ensure concordance on all codes to reduce post-hoc bias. All discrepancies were reviewed and adjusted by the coding team. Descriptive statistics were used to quantitatively report domains, themes, and codes as appropriate.

## Results

**Figure 1.**
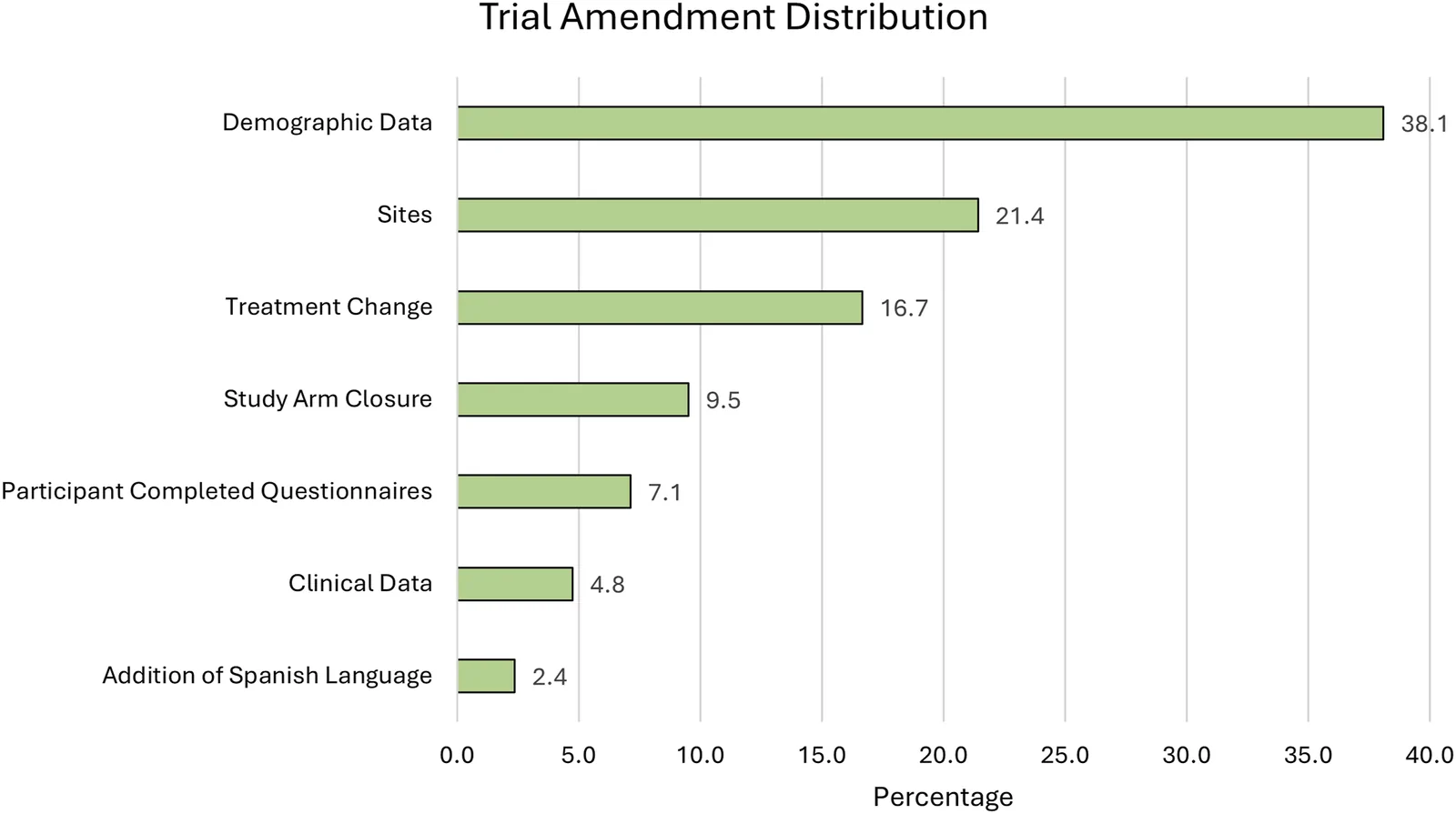
Percent Distribution of Amendment Sub-themes (after Exclusion of Clarifications Category)

S1316 was initiated on March 9, 2015, and accrual (n = 221) was completed on April 27, 2020. Study details can be found in the original S1316 publication.^7^ A total of 37 submissions to the NCI with 133 unique amendments for S1316 were made from 2015 to 2020. We established four domains from the qualitative analysis: Accrual Barriers, Study Design Changes, Data Collection Issues, and Clarifications. Individual themes emerged under each domain (Table 1). Eight percent of the amendments were made for accrual barriers (n = 10), 8% for study design changes (n = 11), 16% for data collection (n = 21) and 68% for clarifications (n = 91). Within clarifications, 48 amendments were categorized as editing (36% of total) while 43 were study protocol related (32% of total). For further sub-analysis, clarifications were excluded as they were comprised mainly of clerical changes with limited applicability towards recommendations concerning trial completion. Themes for Accrual Barriers, Study Design Changes, and Data Collection Issues were further explored. Of these remaining themes, 38% of amendments were for demographic data form revisions (n = 16), 21% for site accrual (n = 9), 17% for treatment change (n = 7), 10% for study arm closure (n = 4), 7% for participant completed questionnaires (n = 3), 5% for clinical data updates (n = 2), and 2% for addition of Spanish language (n = 1, Figure 1).

**Table 1.** Summary of Qualitative Content Analysis Codebook Domains With Their Corresponding Themes and Subsequent Codes

Domains	Themes	Codes
Accrual barriers	1. Sites	1.1 Added
	2. Language	1.2 Removed
		2.1 Spanish Added
Study design changes	3. Treatment change	3.1 Medication change
	4. *Omitted*	3.2 change in design
	5. Study Arm closure	3.3 change in sample size
		3.4 Reactivation
		5.1 study Arm closure
		5.2 study closure
Data collection issues	6. Demographic data	6.1 data collection form Addition
	7. Participant completed questionnaires	6.2 data collection instructions
	8. Clinical data	6.3 Wording
		7.1 Diet Recall
		7.2 Methods of data collection
		8.1 clinical data collection
		8.2 directions
Clarifications	9. Editing	9.1 Protocol editing
	10. Study clarifications	9.2 consent editing
		9.3 data form editing
		10.1 eligibility
		10.2 consent Word choice
		10.3 consent expansion/Clarification
		10.4 Protocol expansion/Clarification
		10.5 Spanish consent
		10.6 Registration clarification
		10.7 Administrative change

## Discussion

### Study Context

Timely completion of S1316 was dependent on the early realization that significant changes needed to be adopted. S1316 was initially a limited site study (six sites) and early accrual after initiation of the study was noted to be problematic for multiple reasons. S1316 was approved prior to the single Institutional Review Board (IRB) reliance policy for all NCTN/NCORP trials through NCI’s central IRB, and several sites experienced delays in IRB approval. This significantly slowed the adoption of S1316 at multiple institutions. For example, at one initial accrual site, senior leadership opted not to participate in the trial. In addition, at another institution, an early potential participant was deemed not eligible as this patient was a Spanish-only speaker. At the time the study was opened, including Spanish-only speaking participants had not been considered and resulted in the loss of multiple potential trial participants. As a result, we initiated study amendments early after the trial was opened to overcome these and several other accrual barriers.

Accrual of patients into the randomized arm of the trial was particularly challenging, as most patients initially opted for the non-randomization arm (“patient choice” pathway).^11^ The initial goal for the randomized arm was 50 patients, which was temporarily reduced to 35 given issues with accrual. We observed that when we closed the non-randomized arms, we were able to improve randomized accrual, allowing eventual completion of full accrual to the randomized pathway. It is unclear if we would have been able to accrue to the randomized arms earlier if the non-randomized arms had not closed, or if the study sites had acquired experience in accruing randomized patients, allowing them to add additional patients to the study.

### Main Findings

Recognizing there were barriers to study accrual, we developed and implemented a standard counseling algorithm for patients to improve participation, particularly within the randomized arms.^11^ Furthermore, accrual amendments facilitating expansion of participating sites from six initial sites to an eventual 30 were pivotal, including international sites, with 78% of patients (173/221) being accrued from non-original study sites. Additionally, with the implementation of the amendment for inclusion of Spanish-speaking patients, 11% of those enrolled in the study were Spanish-speaking participants (25/221); 16 of these patients were from international sites, and nine were from sites in the United States. Once accrual amendments were instituted, the randomized pathway accrual target was increased back to 50 from 35.

S1316 was the first SWOG study to include sites from Latin America in a United States-based trial, leading to logistical issues from the different regulatory and administrative complexities of adding international sites. This caused long delays and initially impeded the ability to accrue to S1316, necessitating crucial amendments for changes of design language to facilitate international sites. This ultimately paved the way for other SWOG studies to routinely evaluate inclusion of Latin American sites. Given our experience, we recommend initial inclusion of Spanish and other non-English speakers in NCTN trials, if possible, to improve trial participation and collaboration with international multicenter institutions, as well as to enroll diverse patients to ensure trial results are generalizable across patient populations.

With regards to Study Design Changes, differences in practice patterns in different facilities necessitated flexibility and resulted in several study amendments. For example, while we were flexible in utilization of somatostatin analogues, these medications were unavailable at several institutions during the study due to difficulty in provision. In addition, it was noted that typical care practice utilized pre-operative total parenteral nutrition at several institutions. This mandated modifying the initial study protocol to allow this delay in the registration timeline, which contributed towards completion of S1316. Furthermore, the study team felt that these clinical conditions enhanced the generalizability and reflected differences in practice patterns among institutions. Thus, early identification of different institutional practice patterns and submission of corresponding amendments to address these differences can be critical for timely continuation of trials.

### Limitations

We acknowledge that this study has certain limitations. Firstly, while coding for the amendments was initially conducted independently by team members and subsequently during team sessions, post-hoc assignment of codes has the potential to introduce bias. Additionally, there were likely administrative and financial costs incurred due to frequent amendments; however, we did not track financial burden as part of our study. With regards to informed consent forms (ICF), certain amendments did require changes, and a subset of patients were likely enrolled using earlier versions of the ICF, however this did not impact the eligibility of any patients. Importantly, no patient needed to re-consent during the trial. Lastly, we attempted to assess the relationship between amendment modifications and accrual, however given delays in amendment approval by the NCI and variable site IRB approvals, a direct correlation could not be examined; nevertheless, as outlined in the results, it was reasonably clear which amendments affected accrual.

### Conclusions

Through this analysis, we highlight the challenges of conducting a trial with both a “patient choice” pathway and randomization pathway. The target sample size for the pilot-sized randomized component (n = 50) was determined not in the usual sense of providing adequate power for testing for differences within the randomized pathway itself. Rather, the sample size was considered the minimum needed to provide a useful, unbiased estimate of the differences between arms (accuracy) while the incorporation of the “patient choice” component in the analyses, corrected for measured confounders, would help to provide power (precision). This pragmatic design was intended to balance the expected difficulties in accruing to the randomized pathway while acknowledging the need for unbiased estimates. In future studies, the potential creation of an algorithm to better focus on the randomized arm could be quite useful. We hope subsequent randomized controlled trials can be done in these patients, now that some level of feasibility has been established via S1316.

S1316 provides a constructive framework to demonstrate that randomized clinical trials for surgical treatment of MBO are feasible, allowing prospective study of patient populations with advanced cancer in the palliative setting. From experience garnered via the S1316 trial, constant and consistent re-appraisal by study leadership led to early amendments which positively enhanced accrual and ultimately, completion of this NCORP trial. Our iterative content analysis of these amendments highlights some of the areas of focus required to improve accrual and aid in quality optimization. Moving forward, future trials may implement strategies we have highlighted from the onset of their study to optimize efficient usage of resources. Furthermore, they may also better anticipate potential amendment adjustments and require implementation of modification strategies at regular intervals during the course of the trial to allow study completion. Despite the challenges posed by studying a heterogeneous patient population by comparing two disparate treatment approaches, we believe that the lessons learned in this international, multi-site clinical trial have practical applications and can help guide future study design.

## Data Availability

The data underlying this article are available in the article; raw data is available upon request from the corresponding author.*

## References

[1] Farooq MS Karakousis GC Krouse RS . Malignant bowel obstruction: historical lessons, current trends, and future directions. Surgical Oncology Insight. 2024;1(2):100046. doi:10.1016/j.soi.2024.100046

[2] Tuca A Guell E Martinez-Losada E Codorniu N . Malignant bowel obstruction in advanced cancer patients: epidemiology, management, and factors influencing spontaneous resolution. Cancer Manag Res. 2012;4:159-169. doi:10.2147/CMAR.S29297 PMC3421464 22904637

[3] Madariaga A Lau J Ghoshal A , et al. MASCC multidisciplinary evidence-based recommendations for the management of malignant bowel obstruction in advanced cancer. Support Care Cancer. 2022;30(6):4711-4728. doi:10.1007/s00520-022-06889-8 PMC9046338 35274188

[4] Cousins SE Tempest E Feuer DJ . Surgery for the resolution of symptoms in malignant bowel obstruction in advanced gynaecological and gastrointestinal cancer. Cochrane Database Syst Rev. 2016;2016(1):CD002764. doi:10.1002/14651858.CD002764.pub2 PMC7101053 26727399

[5] Anthony T Baron T Mercadante S , et al. Report of the clinical protocol committee: development of randomized trials for malignant bowel obstruction. J Pain Symptom Manage. 2007;34(1 Suppl):S49-S59. doi:10.1016/j.jpainsymman.2007.04.011 17544243

[6] Krouse RS Rosenfeld KE Grant M , et al. Palliative care research: issues and opportunities. Cancer Epidemiol Biomarkers Prev. 2004;13(3):337-339. 15006905

[7] Krouse RS Anderson GL Arnold KB , et al. Surgical versus non-surgical management for patients with malignant bowel obstruction (S1316): a pragmatic comparative effectiveness trial. Lancet Gastroenterol Hepatol. 2023;8(10):908-918. doi:10.1016/S2468-1253(23)00191-7 PMC10530384 37541263

[8] Maragh-Bass AC Appelson JR Changoor NR Davis WA Haider AH Morris MA . Prioritizing qualitative research in surgery: a synthesis and analysis of publication trends. Surgery. 2016;160(6):1447-1455. doi:10.1016/j.surg.2016.06.026 27499145

[9] Hsieh HF Shannon SE . Three approaches to qualitative content analysis. Qual Health Res. 2005;15(9):1277-1288. doi:10.1177/1049732305276687 16204405

[10] Vaismoradi M Turunen H Bondas T . Content analysis and thematic analysis: implications for conducting a qualitative descriptive study. Nurs Health Sci. 2013;15(3):398-405. doi:10.1111/nhs.12048 23480423

[11] Deutsch GB Deneve JL Al-Kasspooles MF , et al. Intellectual equipoise and challenges: accruing patients with advanced cancer to a trial randomizing to surgical or nonsurgical management (SWOG S1316). Am J Hosp Palliat Care. 2020;37(1):12-18. doi:10.1177/1049909119851471 PMC6868288 31122027

